# Omalizumab for Severe Asthma: Beyond Allergic Asthma

**DOI:** 10.1155/2018/3254094

**Published:** 2018-09-17

**Authors:** C. C. Loureiro, L. Amaral, J. A. Ferreira, R. Lima, C. Pardal, I. Fernandes, L. Semedo, A. Arrobas

**Affiliations:** ^1^Pulmonology Unit, Hospitais da Universidade de Coimbra, Centro Hospitalar e Universitário de Coimbra, Coimbra, Portugal; ^2^Centre of Pulmonology, Faculty of Medicine, University of Coimbra, Coimbra, Portugal; ^3^Immunoallergology Department, Centro Hospitalar São João, Porto, Portugal; ^4^Immunoallergology Department, Centro Hospitalar de Vila Nova de Gaia/Espinho (CHVNGE), Vila Nova de Gaia, Portugal; ^5^Pulmonology Department, Centro Hospitalar de Vila Nova de Gaia/Espinho (CHVNGE), Vila Nova de Gaia, Portugal; ^6^Pulmonology Department, Hospital Professor Doutor Fernando Fonseca, EPE, Amadora, Portugal; ^7^Pulmonology Department, Hospital São Bernardo, Setúbal, Portugal; ^8^Pulmonology Department, Hospital Santa Marta, Lisboa, Portugal; ^9^NOVA Medical School, Lisbon, Portugal; ^10^Pulmonology Unit, Hospital Geral, Centro Hospitalar e Universitário de Coimbra, Coimbra, Portugal

## Abstract

Different subsets of asthma patients may be recognized according to the exposure trigger and the frequency and severity of clinical signs and symptoms. Regarding the exposure trigger, generally asthma can be classified as allergic (or atopic) and nonallergic (or nonatopic). Allergic and nonallergic asthma are distinguished by the presence or absence of clinical allergic reaction and in vitro IgE response to specific aeroallergens. The mechanisms of allergic asthma have been extensively studied with major advances in the last two decades. Nonallergic asthma is characterized by its apparent independence from allergen exposure and sensitization and a higher degree of severity, but little is known regarding the underlying mechanisms. Clinically, allergic and nonallergic asthma are virtually indistinguishable in exacerbations, although exacerbation following allergen exposure is typical of allergic asthma. Although they both show several distinct clinical phenotypes and different biomarkers, there are no ideal biomarkers to stratify asthma phenotypes and guide therapy in clinical practice. Nevertheless, some biomarkers may be helpful to select subsets of atopic patients which might benefit from biologic agents, such as omalizumab. Patients with severe asthma, uncontrolled besides optimal treatment, notwithstanding nonatopic, may also benefit from omalizumab therapy, although currently there are no randomized double-blind placebo controlled clinical trials to support this suggestion. However, omalizumab discontinuation according to each patient's response to therapy and pharmacoeconomical analysis are questions that remain to be answered.

## 1. Introduction

Asthma is a heterogeneous disease, usually characterized by chronic airway inflammation. It is defined by the history of respiratory symptoms such as wheeze, shortness of breath, chest tightness, and cough that vary over time and in intensity, together with variable expiratory airflow limitation [1].

The prevalence of asthma, one of the most common chronic diseases in the world [2, 3], has increased during the 1970s and 1980s. Epidemiologic studies from the 90s suggested that the prevalence of asthma was around 7.7% in the United States (US)—over 22 million people—and lethality rate was estimated at 5.2 per 100,000 asthmatic patients per year. Worldwide, 200–300 million people suffer from asthma [1–3], and severe asthma comprises 5-10% of all asthmatic patients [4]. In Portugal, the prevalence of asthma is estimated to be of 6.8% [5], affecting around 1 million people. Of these, only 57% have controlled disease, which means that around 300,000 Portuguese asthmatics need a better intervention to control their disease.

The number of hospitalizations due to asthma was 2,728 in 2016, from a total of 262,229 asthmatic patients registered in the Portuguese National Health Service. The standardized mortality rate was, in 2015, of 4.0/100,000 inhabitants for patients above 65 years of age, and of 0.1/100,000 inhabitants for patients below 65 years of age. Nevertheless, and according to the latest Organisation for Economic Co-operation and Development (OECD) report, Portugal is among the countries with less mortality and the country with less hospitalizations due to asthma [6].

The high prevalence of asthma, the impairment of quality of life, the absenteeism, and the large health resources needed to manage this disease makes the economic burden of asthma one of the highest among all chronic diseases. Asthma-related costs have been estimated at up to 2% of the economic cost of all diseases in developed countries [7]. A recent systematic review examined 68 papers on the economic burden of asthma between 1966 and 2008 and concluded that despite the availability of effective preventive therapies, the cost of asthma treatment has increased significantly over the last few decades [8]. A study conducted in Portugal in 2010 concluded that asthma in adults poses a significant economic burden on the Portuguese healthcare system. Total costs amounted to a grand total of €386,197,211.25, with direct costs representing 93% or €359,093,559.82, 2.04% of the total Portuguese healthcare expense in 2010. The major costs were acute care usage (30.7%) and treatment (37.4%). A considerable portion of this burden might be eased by improving asthma control in patients, as uncontrolled patients' costs are more than double those of controlled asthma patients [9].

Severe asthma has a heterogeneous definition. The World Health Organization (WHO) suggests that severe asthma includes three groups: (1) untreated asthma; (2) incorrectly treated asthma (as a result of nonadherence, persistent triggers, or comorbidities); and (3) difficult-to-treat asthma. It is also important to distinguish between severe asthma, comprising patients requiring medium/high doses of inhaled corticosteroids in combination with LABA or other controller, and uncontrolled asthma, resulting from inappropriate therapy or persistent problems with adherence or comorbidities [1]. According to the British Guidelines for Asthma, difficult asthma is defined as that with persistent symptoms and/or frequent asthma attacks despite treatment with high-dose therapies or continuous or frequent use of oral steroids [10]. Untreated patients have been recently omitted in the 2014 revision document produced by the task force of the European Respiratory Society (ERS) and the American Thoracic Society (ATS) [11].

Regarding the exposure trigger, generally asthma can be classified as allergic (or atopic) and nonallergic (or nonatopic or intrinsic) asthma. Allergic and nonallergic asthma are distinguished by the presence or absence of clinical allergic reaction and in vitro IgE response to specific aeroallergens [12, 13]. The triggering of an inflammatory cascade mediated by Immunoglobulin E (IgE) mast cells' activation, with eosinophils and Th2 lymphocyte synthesis, mobilization, and activation in the airways with IL-4, IL-5, and IL-13 production, leads to bronchial constriction and mucus production with airways narrowing [14–21]. The mechanisms of allergic asthma have been extensively studied with major advances happening in the last two decades. Nonallergic asthma is characterized by its apparent independence from allergen exposure and sensitization, but also by a higher degree of severity [12, 13].

Of note, it is important to distinguish nonallergic asthma from aspirin exacerbated respiratory disease (AERD) which also has its own epidemiology, physiopathology, and clinical features: these patients often develop asthma symptoms years after developing rhinitis and nasal polyps due to increased production of cysteinyl–leukotrienes most probably as a result of a polymorphism of the cysteinyl–leukotriene synthase gene [22].

Whether these different clinical subsets of asthma are due to different etiopathogenesis or a different spectrum (or phenotype) of the same disease resulting from different underlying unrecognized mechanisms is still a matter of ongoing debate [15, 23].

This review was prepared and discussed by a group of specialists belonging to the Portuguese Network of Severe Asthma Specialists—REAG.

### 1.1. Allergic versus Nonallergic Asthma

There are similar clinical and physiopathological phenomena between allergic and nonallergic asthma: both can be triggered by exercise, inhaled irritants, or upper airway tract infection; both are associated with rhinitis and both can have higher total serum IgE, airways IgE, airways Th2 cells and Th2, and eosinophilic chemokines and cytokines. Recently, different studies have tried to find a common pathophysiological and immunobiological pattern between both forms of asthma. According to these studies, nonallergic patients may produce the same inflammatory mediators as allergic patients after local IgE production by T lymphocytes at the bronchial and lung mucosal surface where antigens are presented. This was demonstrated comparing bronchial biopsies samples of nonatopic asthma patients, atopic asthma patients, and nonasthmatic controls [12, 24–27].

Clinically, allergic and nonallergic asthma are virtually indistinguishable during exacerbations, since both lead to signs and symptoms of variable lower airways narrowing and obstruction, which is reversible, at least partially, with bronchodilators [14, 18, 20, 21, 28].

By definition, allergic asthma is clearly associated with allergenic triggering, positive skin prick test, and raised specific IgE (sIgE) [15, 23, 29]. On the other hand, nonallergic asthma is usually of late onset, shows no familial patterns and no genetic trends have been recognized [15, 23, 30], has a higher female prevalence, and tends to be of difficult control and with more severe relapses. A patient with asthma is diagnosed with nonallergic asthma if skin prick tests are negative and no circulating sIgE are found [14, 18, 20, 21, 28, 31, 32].

The relationship between allergic and nonallergic asthma prevalence is difficult to ascertain. In some studies, nonallergic asthma prevalence appears to be increasing more than allergic asthma [15]. According to the Swiss Sentinel Surveillance Network (SSSN), the consultations for asthma have decreased over time mainly due to a decrease of allergic asthma. Consultations for nonallergic asthma did not change significantly between 1999 and 2005 [33].

The true prevalence of severe asthma among nonallergic patients compared to allergic asthma patients is uncertain. Most of the studies assume that severe disease is more prevalent among nonatopic asthma patients. There are conflicting data regarding prevalence trends of asthma and atopy over the last 10–15 years [33]. The proportion of asthmatics with severe disease and a negative skin prick test varies from 17 to 34% in the Severe Asthma Research Program (SARP) study [34] to 50% in the ENFUMOSA study [35]. In the ENFUMOSA study, a cross-sectional analysis, it was found that patients with severe asthma were less likely to be skin prick-positive and more likely to have high levels of neutrophils in sputum than patients with less severe asthma [35]. On the other hand, the U-BIOPRED cohort [36] reported a 76.6% incidence of atopy in severe asthma, including nonsmokers, smokers, and ex-smokers.

Although the prevalence and social and financial burdens of nonallergic asthma seem to be lower than in allergic asthma [19], from a clinical point of view, nonallergic asthma is a true challenge: these patients are usually the most difficult to diagnose, due to their specific epidemiologic features, and the most difficult to treat and control.

### 1.2. Phenotypes

There is a complex network of different mechanistic and clinical features which are likely linked by a common pattern of reversible respiratory distress associated to distal airways narrowing. In the last decades efforts have focused on the classification of different subsets of asthma patients according to its epidemiology, immunology, biomarkers, response to specific pharmacotherapies, and long-term prognosis. These are broadly called phenotypes: a set of clinical features of a specific genetic pattern in a specific environment. The main goal of the phenotype and endotype philosophy is the development of targeted and personalized pharmacological approaches. Phenotype definition is particularly important in patients with moderate to severe disease and who are not controlled with usual therapy. A detailed and systematic clinical history, including comorbidities, spirometry with bronchodilator test, a skin or blood test panel for sIgE to common regional airborne allergens, and a peripheral blood eosinophil count are very useful for establishing phenotypes. With this information, allergic and nonallergic asthma and eosinophilic or noneosinophilic asthma can be distinguished. This distinction has prognostic and therapeutic implications.

However, although the above-mentioned four phenotypes are considered to be the major ones, research on asthma phenotypes has increased exponentially in the last years and cluster analysis has identified several distinct clinical phenotypes of asthma [34, 37–39]. There is, nonetheless, a clear heterogeneity regarding asthma phenotypes. GINA considers five phenotypes [1] and Wenzel et al. proposed thirteen in 2006 [40]. However, in 2012, these thirteen phenotypes have been reduced to five, due to the evolution towards linking biology to phenotype, namely, at the molecular and genetic levels [41]. In 2013, Campo et al. [42] proposed 6 severe asthma phenotypes subdivided in clinical and inflammatory phenotypes—Table 1. Smoking is not a phenotype but a disease modifying factor with prognostic implications [42].

### 1.3. Biomarkers

Several biomarkers have been tested for diagnosis and prediction of clinical response to therapy in asthma, with the aim of achieving personalized therapy.

Severe asthma is usually characterized by a type 2 disease, associated with atopy and/or eosinophilic inflammation of the airways [43]. However, inflammation in severe asthma is not always characterized by the presence of eosinophils and cytokines of the high-Th2 endotype; in many cases, it may be low-Th2 neutrophilic or low-Th2 paucigranulocytic (type 1 disease) [42].

Currently there are several biomarkers for severe high-Th2 asthma, but there is a clear need to identify and select biomarkers of the low-Th2 endotypes. However, this is not an easy task, and several studies in severe asthmatics, such as the ENFUMOSA [35], TENOR [44], SARP [34], and, more recently, the U-BIOPRED [36], have shown a remarkable heterogeneity in the clinical presentation and in the underlying pathophysiological mechanisms of severe asthma.

#### 1.3.1. High-Th2 Endotypes

Although heterogeneous, the classification of the high-Th2 endotypes is mainly based on sputum and systemic eosinophilia [45], and this is considered to be a relevant biomarker. These endotypes also show higher epithelial expression of total IgE [15, 44] and Th2 cytokines such as interleukines IL-4, IL-5, and IL-13 [15], two of which, IL-4 and IL13, directly contribute to IgE class switch, thereby increasing IgE [46]. Other known and established biomarkers of Th2 predominant asthma are exaled nitric oxide (FeNO) [47–50] and serum periostin [51]. In a recent study by Busse et al. [52], the authors defined high-Th2 as IgE ≥100 IU/ml, eosinophils count ≥ 300/*μ*l, and FeNO ≥30 ppb. Currently, total IgE and serum eosinophils are used not only as disease biomarkers but also as variables on the treatment algorithm of a specific subgroup of severe asthmatic patients who are eligible for anti-IgE omalizumab [53] or anti-IL5 mepolizumab [54]. Indeed, an analysis of biomarkers of the EXTRA study [55] showed that combining biomarkers on the high-Th2 endotypes had therapeutic response implications: patients with severe atopic asthma with high IgE values and Th2 biomarkers (high blood eosinophils and periostin and high FeNO values) showed a better response to omalizumab therapy.

#### 1.3.2. Low-Th2 Endotypes

Although high-Th2 asthma with atopy and eosinophilia is easy to identify, there is no accepted and consensual definition for the low-Th2 endotypes [56–58], which comprise around one-third of severe asthmatic patients [59].

Low-Th2 endotypes are currently identified in clinical practice as the absence of biomarkers of atopic asthma and/or eosinophilia. In the majority of cases, the low-Th2 endotypes are defined by the absence of Th2 inflammatory biomarkers and characterized as neutrophilic inflammation and, less frequently, by paucigranulocytic inflammation [42, 56].

Although there is no consensus regarding the percentage of sputum neutrophils that would define the neutrophilic asthma phenotype, some reports mention values between 40 and 70% [59].

Beyond the sputum leukocyte content, other specific biomarkers that are able to discriminate high-Th2 from low-Th2 are currently under investigation, but are still not applicable in clinical practice.

IL-8 is a cytokine associated with chemotaxis and neutrophilic degranulation and has been found to be elevated in the sputum of patients with severe resistant asthma [60–62]. CXCR1 and CXCR2 have been also found to be elevated in neutrophilic asthma [62]. Other potential biomarkers of neutrophilic asthma are myeloperoxidase [62] and neutrophilic elastase [61, 62] that can be assessed in sputum of this subgroup of severe asthmatics.

IL-17 is a biomarker of activation of the Th17 pathway, and correlations between the presence of IL-17 and the level of neutrophils in induced sputum and in circulation have been found in patients with severe asthma [62, 63].

There are currently no biomarkers for the subgroup of patients with paucigranulocytic asthma [62]. In this population of patients there is no predominant inflammatory type, and it is possible that other biomarkers of severe asthma, namely, biomarkers of airway remodelling such as osteopontin and angiopoietin, are relevant.

It is necessary to unravel the pathophysiological mechanisms of low-Th2 endotypes in order to identify future biomarkers of these subtypes of asthma [41, 56, 62].

**Figure 1:**
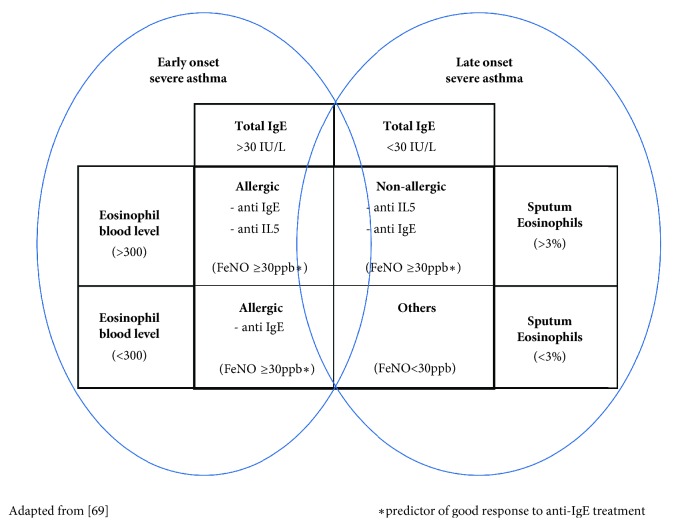
Proposed biomarkers to stratify asthma by phenotypes are still not robust enough to guide therapy in clinical practice.

Currently there are no accurate or precise biomarkers to stratify asthma phenotypes and guide therapy in clinical practice, as illustrated in Figure 1.

### 1.4. Effect of Interaction of Comorbidities

Uncontrolled allergic rhinitis, gastroesophageal reflux disease (GERD), obesity, vitamin D deficiency, noncompliance to therapy, and trigger exposure are among the most important effect modifiers of asthma. Of these, due to its prevalence, obesity is one of the most feared comorbidities in asthma patients.

Obese asthma patients show synergy among the two pathologies, i.e., the complexity of the disease is higher than the sum of the diseases, and this interaction worsens the prognosis. Obesity worsens preexisting asthma, through both biochemical and mechanical effects, and potentially impairs response to treatment, and obese patients are more likely to suffer from nonallergic asthma than nonobese patients [64, 65].

Even in obese asthmatic patients it seems to be possible to distinguish two different clinical courses based on age of onset and Th2 related biomarkers: early-onset asthma tends to have a more atopic disease, higher IgE, and greater bronchial hyperresponsiveness. These patients seem to have allergic asthma that is complicated by obesity. On the other hand, obese patients with late-onset asthma tend to have less atopy, bronchial hyperresponsiveness, and lower levels of Th2 inflammation. These patients have asthma that has developed in the setting of obesity [66].

## 2. Treatment Options for Severe Allergic and Nonallergic Asthma

The aim of therapy in asthma is achieving disease control. Disease control is considered by the British Thoracic Society [10] as

- no daytime symptoms
- no night-time awakening due to asthma
- no need for rescue medication
- no asthma attacks
- no limitations on activity including exercise
- normal lung function (in practical terms FEV1 and/or PEF>80% predicted or best)
- minimal side effects from medication.

The clinical management of nonallergic asthma is similar to that of allergic asthma. It comprises a combination of nonpharmacological approaches, namely, trigger avoidance and control of comorbidities and pharmacological approaches [1, 10, 67]. Pharmacological approach initiates with ICS as the mainstay of therapy with the addition of LABA if this is insufficient to control symptoms [1, 10, 67]. Additional add-on therapy to ICS and LABA according to disease control includes increasing doses of ICS or add-on LAMA, LTRA, or theophylline [1, 10]. Almost 90% of asthma patients can generally be controlled with ICS and LABA. Of the remaining 10%, between 17% and 50% are nonallergic asthma according to the SARP and ENFUMOSA studies [34, 35]. The U-BIOPRED study reported a 30% incidence of nonatopy in the asthma groups [36].

The presence of comorbidities should prompt the initiation of nonpharmacological and pharmacological strategies towards comorbidities, namely, obesity and GERD.

With the breakthrough of monoclonal antibodies (mAbs) therapies on the verge of the 21^st^ century new pharmacological approaches have been developed and tested in these patients [24, 68]. Therapy with mAbs is a specific subset of immunotherapy using passive immunity in which preformed antibodies against a target antigen are injected into the body. MAbs can efficiently target an antigen blocking or initiating a biochemical cascade event and through this mechanism achieve a clinical response [24, 68]. This implies a much higher linkage between pathophysiology, clinical and pharmacotherapy to select the subset of patients who will benefit the most from biological therapy, which revisits phenotypes, immunobiology and endotypes.

### 2.1. Treatment Options in Severe Allergic Asthma

Sputum analysis and FeNO are very useful in predicting Th2 asthma phenotype, even if no eosinophilia is present. This is of utmost importance to therapeutic strategy definition: allergic asthma with elevated eosinophils and FeNO is more likely to respond to ICS [16] and omalizumab [55]. Allergic Th2 phenotype poorly controlled asthmatic patients should be considered good candidates for omalizumab therapy after add-on ICS/LABA/leukotriene/theophylline therapy [69–71].

Omalizumab is a monoclonal antibody designed to bind and inactivate IgE and was approved by EMA in 2009. For patients ≥6 years old omalizumab is indicated as add-on therapy to improve asthma control in patients with severe persistent allergic asthma who have a positive skin test or in vitro reactivity to a perennial aeroallergen and frequent daytime symptoms or night-time awakenings and who have had multiple documented severe asthma exacerbations despite daily high-dose inhaled corticosteroids, plus a long-acting inhaled beta2-agonist. For patients ≥12 years of age a reduced lung function (FEV_1_ <80%) is also required [72].

Omalizumab blocks free serum IgE and limits its binding to the Fc*ε*RI receptor on the surface of mast cells and basophils. This blockade leads to a reduction in the specific inflammatory response induced by activation of effector cells during the encounter with the allergen [73].

Omalizumab has been also demonstrated to reduce the expression of Fc*ε*RI on the surface of circulating mast cells and basophils [74, 75] which results in a decrease in the release of mediators induced by allergenic stimuli* in vitro* and* in vivo* [74, 76, 77]. Omalizumab also seems to intervene in the regulation of the number of circulating basophils which decreases in the treated child [78].

Beyond the anti-IgE mechanism centered on basophils and mast cells, several recent experimental data and clinical observations show that the mechanism of action of omalizumab is more complex than just blocking the allergic response, some of which are mentioned below.

Several studies have shown a decrease in the number of circulating eosinophils and bronchial tissue eosinophils in asthmatics treated with omalizumab [79–82]. Patients with steroid-resistant asthma have been shown to have higher levels of eosinophils, and in these cases omalizumab is a very effective treatment, reducing circulating eosinophils [83]. A proapoptotic effect of omalizumab on eosinophils may contribute to this decrease [84]. Moreover, a study exploring the potential of three biomarkers of Th2-driven inflammation (FeNO, peripheral blood eosinophils, and serum periostin) to predict response to treatment to omalizumab in patients with severe allergic asthma concluded that patients in the high-biomarker subgroup showed a significant decrease in the percentage of exacerbations compared to the low-biomarker subgroup, suggesting that these patients may achieve greater benefit from omalizumab therapy. However, the benefit of such a predictive biomarker of efficacy of omalizumab therapy is currently not established [55].

In a recent study of 673 patients, high levels of periostin and NO exhaled before treatment with omalizumab were associated with a significant decrease in the number of exacerbations [55]. Omalizumab appears to be targeting this Th2 inflammation and a decrease in exhaled NO after treatment has been found in various studies [85]. High levels of these markers prior to initiation of omalizumab have been proposed as biomarkers that predict efficacy with this therapy [55].

Various* in vitro*,* ex vivo*, and/or* in vivo* studies from blood samples, bronchial biopsies, or exhaled air condensates have shown mainly a decrease in the cytokines involved in the recruitment, activation, and survival of eosinophils and IL-5, IL-13, IL-4, IL-8, GM-CSF, eotaxin, RANTES, and the Th2 orientation of the immune response. IFN-*γ*, an anti-inflammatory cytokine, was not modified in two* ex vivo* studies after 16 weeks of treatment with omalizumab [81, 86]. A modulation of the transcription and/or secretion of these different cytokines could thus contribute to a decrease in the recruitment and activation of the inflammatory cells involved in the late inflammatory stage of asthma and reduce long-term remodelling of the airways [87].

In addition to the above, omalizumab has a preventive effect on viral-induced exacerbations in children with allergic asthma, since blocking IgE decreases susceptibility to rhinovirus infections and illness [88]. Dendritic cells play a crucial role in innate immune defence against infections, particularly viral infections [89]. During the respiratory allergic response, dendritic cells ensure the presentation of antigens to T lymphocytes and are also capable of polarizing naïve T lymphocytes in Th2 lymphocytes [90]. Dendritic cells express the Fc*ε*RI receptor on their surface, such as basophils and mast cells [91]. The binding of IgE to dendritic cells inhibits their antiviral capacities [92, 93]. A decrease in the expression of Fc*ε*RI on dendritic cells induced by omalizumab may enhance antiviral immune responses and participate in the prevention of a significant number of asthma exacerbations as demonstrated [88].

### 2.2. Treatment Options in Severe Nonallergic Asthma

Patients with nonallergic asthma are usually more severe and require higher doses of ICS to control symptoms, which may reflect the fact that there may be a degree of corticosteroid resistance as a result of superantigen exposure and activation of MAP kinase pathways [15, 24]. Although patients with severe asthma represent “only” 10% of asthmatic patients, they are the most challenging and with most impairment of quality of life and absenteeism [1, 8, 19].

Severe asthma patients with a non-Th2 phenotype with sputum neutrophilia might benefit from macrolide therapy [16]. A very recent study showed that azithromycin reduced asthma exacerbations in both severe eosinophilic and noneosinophilic asthma, suggesting an immunomodulatory effect of macrolides [94]. This immunomodulatory effect may be a possible mechanism of action of omalizumab in both eosinophilic and noneosinophilic asthma. On the other hand, patients with nonallergic but with clear high-Th2 features might be considered good candidates for biotherapies against IL-5, such as mepolizumab or reslizumab [69–71].

In nonallergic asthma, there is frequent elevation of total IgE, including at the bronchial tissue level [95] and it is now established that dendritic cells participate in its pathophysiology [96, 97]. As in allergic asthma, omalizumab reduces the expression of Fc*ε*RI on the dendritic cells of nonallergic asthma patients [12]. It is likely that other cells expressing Fc*ε*RI involved in the pathophysiology of certain nonallergic asthma phenotypes are targeted by omalizumab [98]. Evidence and especially good quality evidence is emerging regarding the efficacy and safety of off-label uses of omalizumab in severe nonallergic asthma [12, 24, 53, 68, 99–106].

The field of action of omalizumab is therefore not limited to a simple anti-IgE activity. The molecule can inflect airway remodelling on one hand and induce clinical efficacy in nonallergic pathologies, but the mechanisms of action at the cellular and cytokine level, anti-Th2 and anti-inflammation, still need to be clarified. In-depth knowledge of the mechanisms of action of omalizumab would make it possible to identify predictive biomarkers of efficacy, which are valuable in the phenotyping and therapeutic management of patients with severe asthma.

## 3. Conclusions

Although no good quality evidence is currently available to determine which patients with severe nonatopic asthma should be selected for omalizumab treatment, some issues should always be kept in mind: (a) the diagnosis of nonatopic asthma is not easy and should be carefully confirmed; (b) the definition of severe asthma is heterogeneous and should always be carefully assessed; (c) biomarkers may be helpful to select subsets of patients which might benefit from omalizumab treatment; (d) poor adherence and comorbidities, mainly obesity, interact negatively with asthma and should always be addressed with specific pharmacological and nonpharmacological measures. Based on literature and clinical experience of the authors, there is a clear benefit for allergic asthma patients to be treated with omalizumab. Moreover, those patients with severe nonatopic asthma (including those with high FeNO as a marker of IL-13 inflammation, high eosinophils, and periostin), uncontrolled besides optimal nonpharmacological and pharmacological treatment,** may benefit from omalizumab** therapy. However, when to suspend omalizumab according to response to therapy in each patient and pharmacoeconomical analysis are questions that remain to be answered.

## Figures and Tables

**Table 1:** Severe asthma phenotypes proposed by Campo *et al. *[42].

**Clinical phenotypes**	**Characteristics**
Asthma with frequent severe exacerbations	Frequent severe exacerbations with periods of relative stability between exacerbations

Asthma with fixed airflow obstruction	Irreversible persistent and progressive airflow obstruction

Corticosteroid-dependent asthma	Symptoms cannot be controlled, despite high doses of ICS, and patients require daily doses of OCS. Reducing the dose of OCS can often lead to clinical worsening and exacerbations

**Inflammatory phenotypes**	

Persistent severe eosinophilic asthma	Eosinophilia in bronchial biopsies and induced sputum despite high doses of ICS or OCS. Characterized by more symptoms, lower FEV_1_ values, and more severe exacerbations than the non-eosinophilic subtype

Non-eosinophilic severe asthma with increased neutrophils	Eosinophils are either absent from the airway or suppressed by treatment despite the presence of several symptoms, with inflammation of the airway characterized by an increased percentage of neutrophils

Severe paucigranulocytic asthma	It does not involve inflammation by the classical cell types in the bronchial biopsy. Inflammation may be located in the distal airway, which is inaccessible for biopsy, or it may be due to a bronchiolitis-type disease. No thickening of the subepithelial basement membrane or signs of classic inflammation are observed. Other inflammation pathways and other cell types could also be activated

ICS: inhaled corticosteroids; OCS: oral corticosteroids.
